# CagA status & genetic characterization of metronidazole resistant strains of H. pylori from: A region at high risk of gastric cancer

**DOI:** 10.12669/pjms.304.4840

**Published:** 2014

**Authors:** Jin-Yong Yue, Jing Yue, Ming-Yi Wang, Wen-chong Song, Xiao-Zhong Gao

**Affiliations:** 1 Jin-Yong Yue, Department of Gastroenterology, Weihai Municipal Hospital affiliated to Dalian Medical University, Weihai, Shandong, 264200, PR China.; 2 Jing Yue, Department of Gastroenterology, Weihai Municipal Hospital affiliated to Dalian Medical University, Weihai, Shandong, 264200, PR China.; 3 Ming-Yi Wang, Department of Clinical Lab, Weihai Municipal Hospital affiliated to Dalian Medical University, Weihai, Shandong, 264200, PR China.; 4 Wen-chong Song, Department of Gastroenterology, Weihai Municipal Hospital affiliated to Dalian Medical University, Weihai, Shandong, 264200, PR China.; 5 Xiao-Zhong Gao, Department of Gastroenterology, Weihai Municipal Hospital affiliated to Dalian Medical University, Weihai, Shandong, 264200, PR China.

**Keywords:** Helicobacter pylori, Metronidazole resistance, Integrons, Plasmid, cagA status

## Abstract

***Objective:*** The aim of study was to determine relationship between* cagA* and genetic characterization of metronidazole (MTZ) resistant *H. pylori* strains from a region at high risk of gastric cancer.

***Methods:*** 172 *H. pylori* strains were isolated from the patients with dyspeptic symptoms, and antimicrobial susceptibility testing for MTZ was assessed by E-test. *rdxA* and *frxA* genes were amplified using PCR among the MTZ resistant isolates. The status of the plasmid and classes 1～3 integrons were investigated in all isolates.

***Results: ***MTZ was detected in 88 isolates (51.16%). Variations in the *rdxA* gene leading to alterations of amino acids in RdxA proteins were identified in all MTZ resistant strains. FrxA contained missense alterations in 55 MTZ resistant isolates, while the premature truncation of FrxA was caused by frameshift mutations in 9 MTZ resistant strains. Plasmid was found in one MTZ sensitive strain (0.58%), and none of Class 1～3 integrases gene was detected in the studied isolates. The conservative *cagA *fragment was obtained from all clinical isolates of* H. pylori*. The sequence of *cagA* 3' variable region in 164 strains were obtained, including East Asian-type (122, 74.39%) and Western-type (42, 25.61%). Prevalence of Western-type *cagA* 3' variable region was significantly higher in MTZ resistant (33.73%, 28/83) than those of MTZ-sensitive strains (17.28%, 14/81) (*p=0.02*).

***Conclusion:*** A high prevalence of MTZ resistance was found in the region, and bacterial chromosome mutations in the *rdxA *and* frxA *gene still contribute to the high-level MTZ resistance. *H. pylori *strains characterized with West-type *cagA *3’ variable region tend to acquire MTZ resistance in the region.

## INTRODUCTION

The discovery that Helicobacter pylori (H. pylori) were identified as an infectious agent responsible for most gastroduodental diseases has been a major breakthrough in the gastroenterology.^1^^,^^2^ Triple therapy, including two antibiotics and a proton pump inhibitor, has been recommended as the treatment for H. pylori eradication for many years, but the bacterial resistance to one of the most effective antibiotics, metronidazole, is a serious and increasing problem.^3^

The resistant mechanism of *H. pylori* is still a complex problem. Overwhelming evidence indicates that antimicrobial resistance in *H. pylori *is the consequence of mutations located on the bacterial chromosome.^4^^,^^5^ However, genetic exchanges seem to be numerous between different strains of *H. pylori* and therefore the possibility of transmission of resistance does exist.^4^ Plasmid has also been identified in* H. pylor*i, and conjugation can contribute to DNA transfer between clinical isolates.^6^^,^^7^ Integrons are widely distributed among bacteria, which are strongly associated with resistance in plasmids and/or bacterial chromosome. The presence of the class 2 integron was found in 15 of 40 different *H. pylor*i strains in a previous study.^8^

Although the high rate of East Asian-type *cagA*, intact* cag*PAI, virulent *vacA* genotypes, and the intact long-type *dupA* in *H. pylori* was reported in a littoral region of Northeast China with the high risk of gastric cancer,^9^ there is currently no report about the relationship between virulence marker and genetic characterization of antimicrobial resistant strains of *H. pylori*. This study was conducted to evaluate the* cagA* status and genetic characterization of MTZ resistant strains of *H. pylori* in this region.

## METHODS

***Patients & sampling: ***Biopsy samples were taken from the patients, who underwent upper gastrointestinal endoscopy because of dyspeptic symptoms at *Weihai Municipal Hospital affiliated to Dalian Medical University* from June 2010 to June 2013. No patient underwent antimicrobial therapy, proton pump inhibitors (PPIs), or non-steroidal anti-inflammatory drugs a month before their inclusion in the study.

Written and informed consent was obtained from all patients, and the study was conducted upon approval by the Ethical Committee of Weihai Municipal Hospital affiliated to Dalian Medical University.

***H. pylori cultivation & Identification: ***After biopsies were taken, samples were collected in brain heart infusion broth (Oxoid, United Kingdom), and dispersed using a sterile tissue homogenizer within two hours of collection. Every homogenate was inoculated onto Campylobacter agar (Oxoid, United Kingdom) with 8% sheep blood, and *H. pylor*i selective supplement (Oxoid, United Kingdom) in microaerophilic condition (5% O_2_, 10%CO_2_ and 85% N_2_) at 37℃ for 72h. Small dew drop colonies were selected for the identification of *H. pylori* with the phenotypic characteristics and PCR based on 16SrRNA gene sequence as described previously.^9^^,^^10^

***Antimicrobial susceptibility testing: ***The susceptibility of *H. pylori *strains to MTZ was assessed by *E-*test (BioMerieux, France). Bacterial suspensions were prepared in 0.85% NaCl to turbidity of 2.0 McFarland units, and were spread on Mueller-Hinton agar containing 5% old sheep blood within 15 minutes after the preparation. Then, *E-*test stripe was placed on the plates to incubate in microaerophilic conditions at 37℃ for 72h. The assay was strictly performed and results were interpreted according to standard criteria,^11^^,^^12^ and all tests were performed thrice.

***Genomic DNA & plasmid DNA extraction: ***After bacteria were harvested, genomic DNA and plasmid DNA were isolated in all isolates by using bacterial genomic DNA extraction kit (DV810A, Takara) and Plasmid Purification Kit (D821A, Takara), respectively. The assay was strictly performed according to manufacture’s protocol. Extractions were electrophoresed in 1.0% agarose gel and visualized by staining with ethidium bromide under short UV light.

***PCR amplification & cloning of rdxA & frxA genes: ***The *rdxA* and *frxA* genes were amplified in MTZ resistant isolates using PCR with the specific primers (Table-I) as previously described,^13^

The following protocol was used to amplify the two genes: initial denaturation for 5 min at 94℃; 30 cycles of 94℃ for 1min, 52℃ for 35 s, and 72℃ for 1 min; and a final elongation for 10 min at 72℃. After PCR amplification, the amplified PCR products were electrophoresed in 2% agarose gels and examined under UV illumination. DNA sequencing of the amplicon was determined by Life Technologies Corporation.

***PCR for analyzing classes 1, 2 and 3 integrons status in H. pylori: ***The presence of classes 1, 2 and 3 integrons was analyzed in all isolates obtained in this study by using genomic DNA extraction. Three pair primers were used to obtain the converse regions sequence of the three classes integrase genes respectively, while the variable region of the integrons were determined with another pair primers.^14^ Using the internal primers for the class 2 integrase gene, amplification reaction to detect the presence of class 2 integron was carried out as previously described.^8^ All primers are listed in Table-I. Some amplicons were sequenced by Life Technologies Corporation.

***Analysis of cagA status in H. pylori: ***PCR analyses were carried out to determine the presence or absence of *cagA* and detect *cagA* 3’ variable region of *H. pylori *strains as described previously.^9^ After PCR, the amplified PCR products were electrophoresed in 2% agarose gels and examined under UV illumination. All amplicons of *cagA* 3’ variable region were sequenced by Life Technologies Corporation. According to nucleotide analysis,^9^ *cagA* 3’ variable region of the strains fell into two types: East Asian–type and Western–type.

## RESULTS

Five hundred and seventy-one biopsy samples were taken from the patients with upper gastrointestinal diseases, and a total of 172 (30.12%) samples were *H. pylori *culture positive. Antimicrobial susceptibility testing of *H. pylori *strains were implements in vitro. Resistance to MTZ was detected in 88 isolates (51.16%) in this study.

Compared with the sequences of *H. pylori* reference strains J99 and 2669, variations in the* rdxA* gene leading to alterations of amino acids in RdxA proteins were identified in all MTZ resistant strains. Six MTZ resistant strains had the premature truncation of RdxA at position 32, 64, 75, 82, 112, and 137 by *rdxA* mutations which introduced stop codons; thirty-six demonstrated amino acid substitutions induced by nucleotide substitutions; forty-six others showed both nonsense mutations and amino acid substitutions.

**Table-I:** Oligonucleotide primers used in the PCR assay

***Primer***	***Nucleotide sequence (5’-3’)***		***PCR target***	***Expected size (bp)***	***Reference***
*rdxA*-F	GCAGGAGCATCAGATAGTTCT			886	[13]
*rdxA*-R	GGGATTTTATTGTATGCTACAA				
*frxA*-F	GGATATGGCAGCCGTTTATCATT			780	[13]
*frxA*-R	GAATAGGCATCATTTAAGAGATTA				
IntI1F	ACGAGCGCAAGGTTTCGGT		Class 1 integrase gene	565	[14]
IntI1R	GAAAGGTCTGGTCATACATG				
IntI2F	GTGCAACGCATTTTGCAGG		Class 2 integrase gene	403	[14]
IntI2R	CAACGGAGTCATGCAGATG				
IntI3F	CATTTGTGTTGTGGACGGC		Class 3 integrase gene	717	[14]
IntI3R	GACAGATACGTGTTTGGCAA				
5'-CS	GGCATCCAAGCAGCAAG		Variable region of integrons	Uncertain	[14]
3'-CS	AAGCAGACTTGACCTGAT				
Inti2F	GCAAATGAAGTGCAACGC		Class 2 integrase gene	449	[8]
Inti2R	ACACGCTTGCTAACGATG				
*cagA *conservative region		GATAGGGATAACAGGCAAGC		297	[9]
GGGGGTTGTATGATATTTTC					
*tcagA *3’variable region		GGAACCCTAGTCGGTAATG		Uncertain	[9]
GCTTTAGCTTCTGATACC					

The premature truncation of FrxA in nine MTZ resistant strains was caused by frameshift mutations due to nucleotide deletion, including one strain at position 54 as previously reported.^15^ In fifty-five MTZ resistant isolates FrxA contained missense alterations induced by nucleotide substitutions. Nine MTZ resistant strains exhibited no amino acid substitution with nucleotide substitutions (nonsense mutation). For fifteen isolate, there were no specific changes in the frxA gene as compared with variations in those from reference strains.

When all the 172 *H. pylori *isolates were screened for plasmids by electrophoresis, one isolate (0.58%) without antimicrobial resistance was found to harbor plasmid. None of Class 1~3 integrases gene was detected in the studied isolates using the primers, including the internal primers for Class 2 integron. The integrons variable region analysis identified the 172 isolates, and 8 kinds of sequence were found by PCR and sequencing them. The sequences of amplicons were compared with deposited sequences in the GenBank nucleotide sequence database, and were identified as non-specific amplicon.

The expected 297bp PCR product of the conservative *cagA* fragment was obtained from all clinical isolates of *H. pylori*. *cagA* 3’ variable region of 172 strains was amplified, and the sequence of 164 strains were obtained, including East Asian-type (122 strains, 74.39%) and Western-type (42 strains, 25.61%). Prevalence of Western-type *cagA* 3’ variable region was significantly higher in MTZ resistant (33.73%, 28/83) than those of MTZ-sensitive strains (17.28%, 14/81) (*p *=0.02).

## DISCUSSION

Antimicrobial resistance in* H. pylori *is a global problem, which is the main reason for the failure of *H. pylori *eradication. We analyzed the susceptibility of 172 *H. pylori *strains to MTZ isolated from the patients with dyspeptic symptoms.

MTZ resistance rates of *H. pylori *are from 50% to 100% in developing countries. In the study we reported the resistance to it was 51.16%, which was higher than that in some certain areas and countries,^16^^,^^17^ but lower than that in Iran (95%) and Egypt (100%).^18^^,^^19^

The resistant mechanism of MTZ in *H. pylori* is based on the mutations of *rdxA* and *frxA* genes as reported,^15^ and the patterns of variation in* rdxA* and *frxA* genes were highly diversified. Meanwhile, plasmids frequently carry antibiotic resistance encoding genes in many pathogenic bacteria, and different positivity for plasmids in fresh clinical isolates of *H .pylori *has been reported subsequently.^20^^,^^21^

Virtually nothing is known about the significance of plasmid presence in *H. pylori *including their possible role in the antimicrobial resistance. Our study showed that the carrier rate of plasmid in *H. pylori *was lower (0.58%) than in other region.^20^ Integrons are specialized genetic elements frequently carrying gene cassettes mainly encoded antibiotics resistance genes, usually located on plasmids. There are three classes integrons involved in bacterial resistance, and class 2 integron emerges with high carrier rate (37.5%) in clinical isolates of *H. pylori* as reported.^8^ In this study we have analyzed the presence of class 1, 2 and 3 integrons from 172 *H. pylori *strains isolated from the gastric mucosa of Chinese patients. None of Class 1～3 integrases gene was detected in the studied isolates, even though in the isolate with the plasmid.

cagA-negative H. pylori strains tend to acquire MTZ resistance in vitro, and absence of cagA might be a risk factor in development of MTZ resistance.^22^ As previously reported,^9^ all of H. pylori isolated from the region posses with cagA-positive strains and cagA 3’ variable region of most H. pylori strains was East Asian-type in this region. We believe that the variation of cagA 3’ variable region in the region is best explained by genetic recombination. Intriguingly, most H. pylori with Western-type cagA 3’ variable region were MTZ resistant in the study. The mechanism for this might be explained by high activity of the genetic recombination in H. pylori with West-type cagA 3’ variable region.

In conclusion, a high frequency of MTZ resistance in *H. pylori *strains was found in the region at high risk of gastric cancer. Bacterial chromosome mutations in the *rdxA *and* frxA *gene still contribute to the high-level MTZ resistance. Most *H. pylori *with Western-type *cagA* 3’ variable region were MTZ resistant in the region. It indicates that *H. pylori *strains characterized with West-type *cagA *3’ variable region tend to acquire MTZ resistance in the region.

## Notes

***Disclosure of interest: ***The authors declare that they have no conflicts of interest.
